# Inhibition of DNA Methylation Suppresses Intestinal Tumor Organoids by Inducing an Anti-Viral Response

**DOI:** 10.1038/srep25311

**Published:** 2016-05-04

**Authors:** Yoshimasa Saito, Toshiaki Nakaoka, Kasumi Sakai, Toshihide Muramatsu, Kohta Toshimitsu, Masaki Kimura, Takanori Kanai, Toshiro Sato, Hidetsugu Saito

**Affiliations:** 1 Division of Pharmacotherapeutics, Keio University Faculty of Pharmacy, 1-5-30 Shibakoen, Minato-ku, Tokyo 105-8512, Japan; 2 Division of Gastroenterology and Hepatology, Department of Internal Medicine, Keio University School of Medicine, 35 Shinanomachi, Shinjuku-ku, Tokyo 160-8582, Japan

## Abstract

Recent studies have proposed that the major anti-tumor effect of DNA methylation inhibitors is induction of interferon-responsive genes via dsRNAs-containing endogenous retroviruses. Recently, a 3D culture system for stem cells known as organoid culture has been developed. Lgr5-positive stem cells form organoids that closely recapitulate the properties of original tissues. To investigate the effect of DNA demethylation on tumor organoids, we have established organoids from intestinal tumors of *Apc*^*Min/*+^ *(Min)* mice and subjected them to 5-aza-2′-deoxycytidine (5-Aza-CdR) treatment and *Dnmt1* knockdown. DNA demethylation induced by 5-Aza-CdR treatment and *Dnmt1* knockdown significantly reduced the cell proliferation of the tumor organoids. Microarray analyses of the tumor organoids after 5-Aza-CdR treatment and *Dnmt1* knockdown revealed that interferon-responsive genes were activated by DNA demethylation. Gene ontology and pathway analyses clearly demonstrated that these genes activated by DNA demethylation are involved in the anti-viral response. These findings indicate that DNA demethylation suppresses the proliferation of intestinal tumor organoids by inducing an anti-viral response including activation of interferon-responsive genes. Treatment with DNA methylation inhibitors to activate a growth-inhibiting immune response may be an effective therapeutic approach for colon cancers.

Aberrant DNA methylation at CpG island promoters of tumor suppressor genes is frequently observed in various human malignancies^1^. The DNA methylation inhibitor 5-aza-2′-deoxycytidine (5-Aza-CdR), which is an analog of cytidine, has been widely studied and was recently approved for the treatment of myelodysplastic syndrome (MDS)^1^,^2^. Other DNA methylation inhibitors are also being assessed in clinical trials. Although it is believed that the anti-tumor effect of DNA methylation inhibitors is mediated by re-activation of epigenetically silenced tumor suppressor genes in cancer cells, recent studies have proposed that the major effect of DNA methylation inhibitors is induction of interferon-responsive genes via dsRNA-containing endogenous retroviruses (ERVs)^3^,^4^. These findings may represent a major shift in our understanding of the anti-tumor mechanisms of DNA demethylating agents.

Tumor stem cells with self-renewal and multipotential capacity play critical roles in malignant tumors that show a marked capacity for metastasis and invasion. The development of chemotherapy targeting tumor stem cells has important implications for the management of various cancers. However, little is known about the effect of DNA demethylating agents on the proliferation of tumor stem cells. Lgr5, a member of the Wnt signaling pathway, has been identified as a new molecular marker of stem cells in the small intestine, colon, stomach, liver and pancreas^5^,^6^,^7^,^8^,^9^,^10^. A newly developed 3D culture system allows Lgr5-positive stem cells to form budding cyst-like structures (organoids) that resemble the properties of original tissues^5^. This type of 3D culture uses serum-free medium that includes only specifically defined factors such as R-spondin 1 (Rspo1), epidermal growth factor (EGF) and noggin. Rspo1 has been identified as a ligand for Lgr5 and an essential factor for activation of the Wnt signaling pathway^11^,^12^. In addition, a recent study has clearly demonstrated that tumor organoids closely recapitulate the properties of original tumors^13^, suggesting that patient-derived organoids may allow high-throughput drug screening and personalized cancer therapy. To investigate the effect of DNA demethylation on tumor organoids, we established intestinal tumor organoids derived from tumors of *Apc*^*Min/*+^ (*Min*) mice and subjected them to treatment with 5-Aza-CdR and knockdown of *Dnmt1*.

## Materials and Methods

### Animals and experimental design

**Figure 1:**
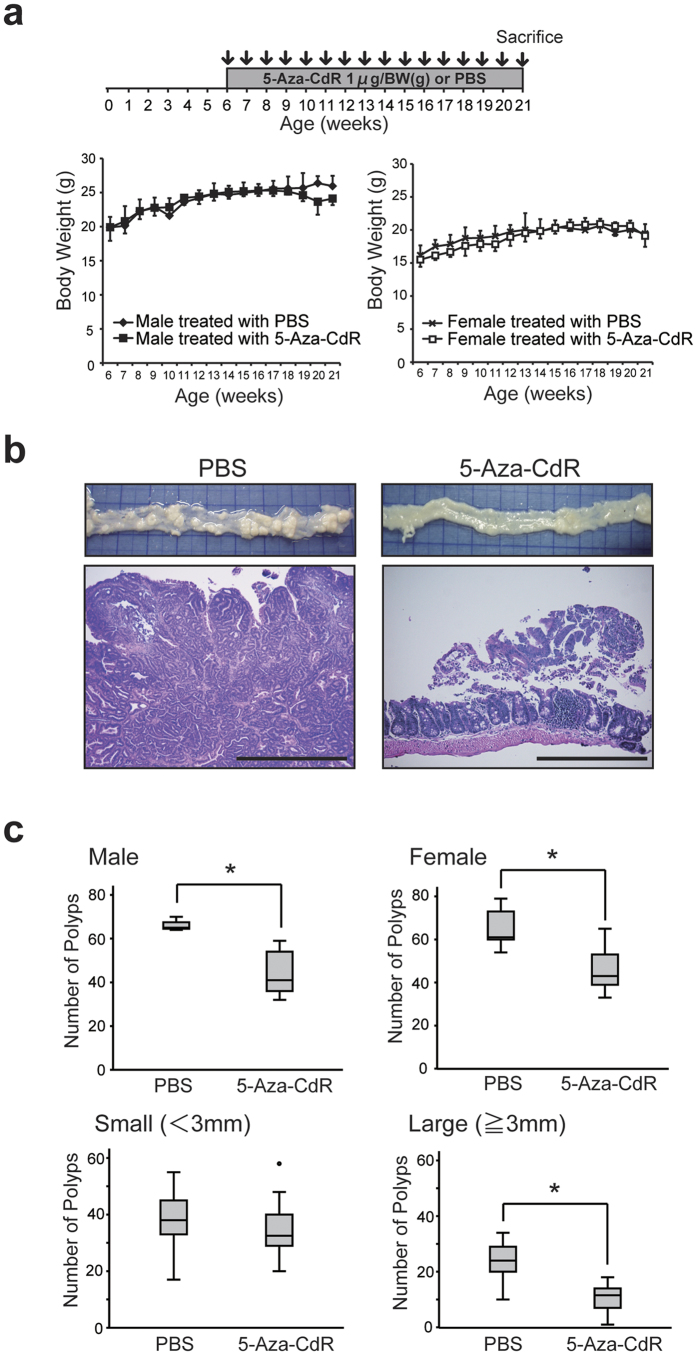
Anti-tumor effect of 5-Aza-CdR on intestinal tumor formation in *Min* mice. (**a**) Experimental design of the animal study and growth curves of male and female *Min* mice. From 6 weeks of age, 1 μg per body weight (g) 5-Aza-CdR or PBS was injected subcutaneously into *Min* mice once a week for 100 days (15 injections). The mice were then dissected at 21 weeks of age, and the number of polyps was counted. (**b**) Examples of the macroscopic and microscopic (HE staining) features of the intestine in *Min* mice treated with either 5-Aza-CdR or PBS. Scale bars: 1000 μm. (**c**) Average numbers of polyps in the intestine of male and female *Min* mice treated with either 5-Aza-CdR or PBS (upper). Average numbers of large polyps (>3 mm) and small polyps (<3 mm) in the intestine of *Min* mice treated with either 5-Aza-CdR or PBS (lower). *p < 0.05.

The experimental design for our animal study is shown in Fig. 1a. All experiments and procedures were approved by the Keio University Animal Research Committee, and all methods were carried out in accordance with the approved guidelines. *Min* mice were maintained in a temperature-controlled specific pathogen-free animal facility and given free access to water and food. They were treated with 5-Aza-CdR (Sigma-Aldrich, 1 μg/body weight, n = 12) or phosphate-buffered saline (PBS, n = 11) by subcutaneous weekly injection. The injections were started at 6 weeks of age and continued for 100 days (15 injections). The weights of the mice were monitored weekly. At 21 weeks of age, the mice were dissected and their intestinal polyps were counted.

### Polyp analysis

The gastrointestinal tract (duodenum to rectum) of *Min* mice was removed immediately after sacrifice, washed with PBS, cut longitudinally and rinsed with PBS, and then fixed with Davidson’s fixative. The number and size of the polyps along the entire length of the intestine were analyzed by investigators who were blinded to the treatment regime used for the mice. Tissues were fixed in buffered formalin, embedded in paraffin blocks, and then processed for histologic analyses using standard protocols with HE staining.

### Establishment of intestinal tumor organoids and 5-Aza-CdR treatment

Isolation and dissociation of stem cells from intestinal tumors of *Min* mice and normal epithelia from wild-type C57BL/6 mice were performed as described previously^8^. Isolated tumor cells and epithelial cells were embedded in Matrigel on ice (growth factor-reduced, phenol red-free; BD Biosciences) and seeded in 48-well plates. The Matrigel was polymerized for 10 minutes at 37 °C, and overlaid with 250 μL/well basal culture medium (advanced Dulbecco’s modified Eagle medium/F12 supplemented with penicillin/streptomycin, 10 mmol/L HEPES, Glutamax, 1 × N2, 1 × B27 [all from Thermo Fisher Scientific], and 1 mmol/L *N*-acetylcysteine [Sigma-Aldrich]) containing the following optimized growth factor combinations: epidermal growth factor (EGF) and noggin for intestinal tumors; EGF, noggin and Rspo1 for intestinal epithelia. Intestinal tumor organoids were treated with 1 or 3 μM 5-Aza-CdR. Twenty-four hours after treatment, 5-Aza-CdR was removed from the culture medium, and regular culture medium was employed thereafter.

### Lentivirus-mediated knockdown of Dnmt1 in tumor organoids

Lentivirus-mediated shRNA against the mouse *Dnmt1* gene (GIPZ lentiviral shRNA) and a shRNA control (GIPZ lentiviral empty vector shRNA) were purchased from Thermo Fisher Scientific. Preparation of organoids and lentiviral infection were performed as described previously^14^. Twenty-four hours after transduction, the infected organoids were selected with puromycin (1 μg/ml).

### Cell proliferation assay

Cell proliferation activity of tumor organoids was examined by WST assay using the Cell Counting Kit-8 (Dojindo, Japan) in accordance with the manufacturer’s instructions. Absorbance at 450 nm was measured using an Infinite M1000 PRO microplate reader (TECAN).

### RNA extraction and microarray analyses

Total RNAs from cultured tumor organoids were extracted using a mirVana isolation kit (Thermo Fisher Scientific). Microarray analyses were conducted by Toray Industries (www.toray.com: Tokyo, Japan). All data were submitted to the GEO database, under the accession number GSE72768. Gene Ontology (GO) and KEGG (Kyoto Encyclopedia of Genes and Genomes) pathway analyses of the microarray data were performed using Genecoids (http://genecoids.dacya.ucm.es/analysis/). Hypergenometric *p*-values corrected by the false discovery rate (FDR) were calculated. The threshold for the significant GO and pathway was set at *p* < 0.05.

### Quantitative RT-PCR

Levels of *Dnmt1* expression were analyzed by quantitative RT-PCR using the TaqMan gene expression assay (Thermo Fisher Scientific) in accordance with the manufacturer’s instructions. Expression levels of interferon-responsive genes (*Oas1, Irf7, Isg15, Rig1, Mda5*) and murine ERVs (IAP-1, MLV, LV30-2, MuRRS) were analyzed by quantitative RT-PCR using the Universal SYBR Select Master Mix (Thermo Fisher Scientific) as described previously^15^,^16^. The primer sequences are summarized in Supplementary Table S1.

### Statistical analysis

Data obtained from polyp counting, WST assay and quantitative RT-PCR were analyzed by independent t-test using the SPSS 23.0 statistical software package. Differences at *p* < 0.05 were considered significant.

## Results

### Anti-tumor effect of 5-Aza-CdR on intestinal tumor formation in *Min* mice

To study the effects of DNA methylation inhibitor on intestinal tumor formation *in vivo*, we treated *Min* mice with 5-Aza-CdR. The experimental design of this study is shown in Fig. 1a. From 6 weeks of age, 1 μg per body weight (g) 5-Aza-CdR was injected subcutaneously into *Min* mice once a week for 100 days (15 injections). Control *Min* mice received PBS instead of 5-Aza-CdR. The mice were then dissected at 21 weeks of age, and the polyps were counted to investigate the suppressive effects of 5-Aza-CdR on tumor progression. Growth curves of male and female *Min* mice treated with either 5-Aza-CdR or PBS are shown in Fig. 1a. As can be seen, treatment with 5-Aza-CdR did not have any significant effect on body weight.

The number of intestinal polyps at 21 weeks of age was determined by examining the mucosa over the entire length of the intestine. Figure 1b shows examples of the macroscopic and microscopic (HE staining) features of the intestine in *Min* mice treated with either 5-Aza-CdR or PBS. Numerous adenomas were found throughout the entire intestine of control *Min* mice treated with PBS, whereas *Min* mice treated with 5-Aza-CdR showed a decrease of adenoma formation. The graphs shown in Fig. 1c demonstrate the number of polyps in male and female *Min* mice treated with either 5-Aza-CdR or PBS. Treatment of male and female *Min* mice with 5-Aza-CdR significantly reduced the average number of intestinal adenomas from 66 to 44, and from 65 to 47, respectively. In addition, the average number of large adenomas (>3 mm) in *Min* mice treated with 5-Aza-CdR was significantly decreased from 24 to11, whereas there was no significant difference in the average number of small adenomas (<3 mm). These results indicate that treatment of *Min* mice with the DNA methylation inhibitor 5-Aza-CdR suppresses the development of intestinal tumors.

### Anti-tumor effect of 5-Aza-CdR on growth of intestinal tumor organoids

**Figure 2:**
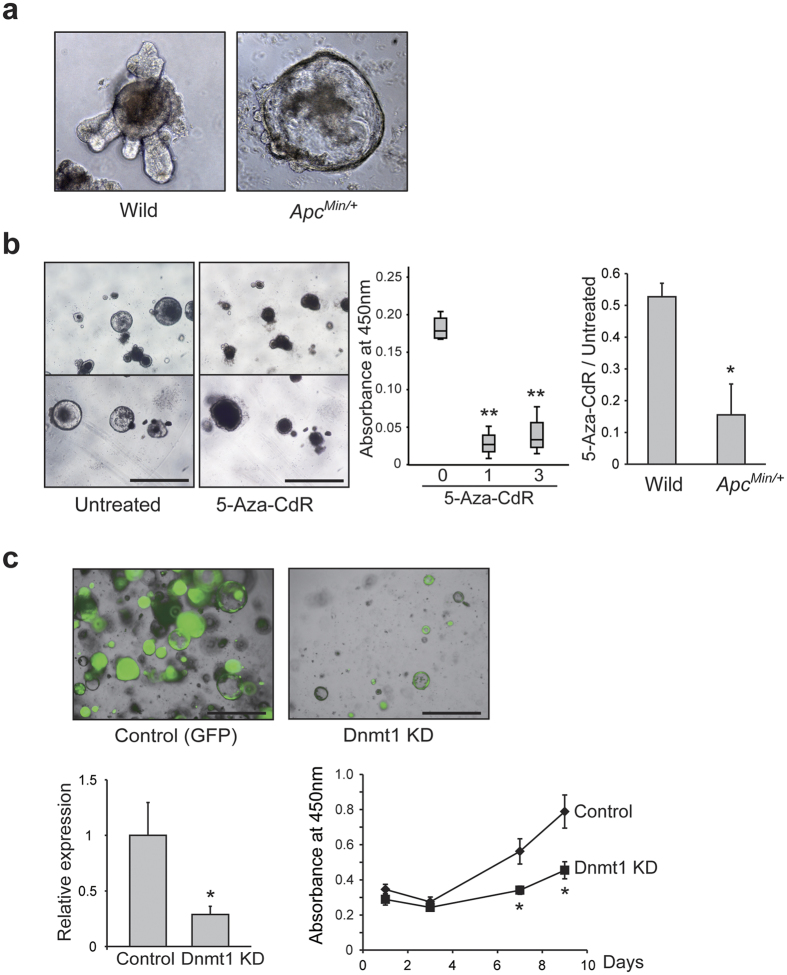
Anti-tumor effect of 5-Aza-CdR treatment and *Dnmt1* knockdown in intestinal tumor organoids. (**a**) Bright-field images of organoids derived from intestinal epithelia of wild-type C57BL/6 mice (Wild) and intestinal tumors of *Min* mice (*Apc*^*Min/*+^). (**b**) Bright-field images of intestinal tumor organoids of *Min* mice after treatment with 5-Aza-CdR. Scale bars: 1000 μm (left). WST cell proliferation assay of intestinal tumor organoids of *Min* mice after treatment with 1 μM or 3 μM 5-Aza-CdR. **p < 0.001 compared to untreated control (middle). WST cell proliferation assay of organoids derived from intestinal epithelia of wild-type C57BL/6 mice (Wild) and intestinal tumors of *Min* mice (*Apc*^*Min/*+^) after treatment with 1 μM 5-Aza-CdR. Values were normalized to untreated controls. *p < 0.005 compared to Wild (right). We analyzed the WST cell proliferation assay of the organoids at 4 days after 5-Aza-CdR treatment. (**c**) Images of intestinal tumor organoids transfected with lentiviral shRNA against *Dnmt1* and control shRNA. Transfection of lentivirus vectors was confirmed by GFP expression in the organoids (upper). Relative expression of *Dnmt1* normalized to *Gapdh* and WST cell proliferation assay in intestinal tumor organoids transfected with lentiviral shRNA against *Dnmt1* and control shRNA. We seeded the cells obtained from the organoids of *Dnmt1* knockdown and control (GFP) at 2 × 10^4^ cells per well, and then examined cell proliferation until 9 days after cell plating. *p < 0.05 compared to control (lower).

To investigate the effects of the DNA methylation inhibitor on intestinal tumor cells *in vitro*, we established cultured organoids from intestinal tumors of *Min* mice as well as intestinal epithelia of wild-type C57BL/6 mice. As described previously^8^, organoids derived from *Apc*-deficient tumors formed cystic organoid structures without the “budding” that is observed in intestinal epithelial organoids derived from wild-type C57BL/6 mice (Fig. 2a). Since Apc loss constitutively activates the Wnt pathway, Rspo1 was dispensable for the culture of *Apc*-deficient tumor organoids. We treated *Apc*-deficient intestinal tumor organoids with 5-Aza-CdR and analyzed the WST cell proliferation assay of the organoids at 4 days after 5-Aza-CdR treatment. As shown in Fig. 2b, the organoids were unable to proliferate after treatment with 5-Aza-CdR. The results of the WST assay clearly indicated that cell proliferation activity of the tumor organoids was significantly reduced after treatment with both 1 μM and 3 μM 5-Aza-CdR. In addition, the cell proliferation activity after treatment with 1 μM 5-Aza-CdR was markedly lower in organoids derived from intestinal tumors of *Min* mice than in organoids derived from normal intestinal epithelia of wild-type mice (Fig. 2b). These results indicate that 5-Aza-CdR exerts an anti-tumor effect on tumor organoids but is much less toxic to normal intestinal organoids.

### Anti-tumor effect of Dnmt1 knockdown on growth of intestinal tumor organoids

The cellular toxicity of 5-Aza-CdR may affect gene expression and proliferation of intestinal tumor organoids. Therefore, to evaluate the effect of DNA demethylation in the absence of cellular toxicity, we next performed lentivirus-mediated knockdown of the major DNA methyltransferase, *Dnmt1*, in intestinal tumor organoids. Successful transfection of lentiviral shRNA against *Dnmt1* and control shRNA was confirmed by expression of GFP in the organoids (Fig. 2c). WST assay was performed to investigate the cell proliferation activity of intestinal tumor organoids. We seeded the cells obtained from the organoids of *Dnmt1* knockdown and control (GFP) at 2 × 10^4^ cells per well, and then examined cell proliferation until 9 days after cell plating. As shown in Fig. 2c, cell proliferation activity of *Dnmt1* knockdown organoids was significantly suppressed in comparison with the control after 7 and 9 days of culture.

### Treatment with 5-Aza-CdR and knockdown of Dnmt1 induce an anti-viral response in intestinal tumor organoids

**Figure 3:**
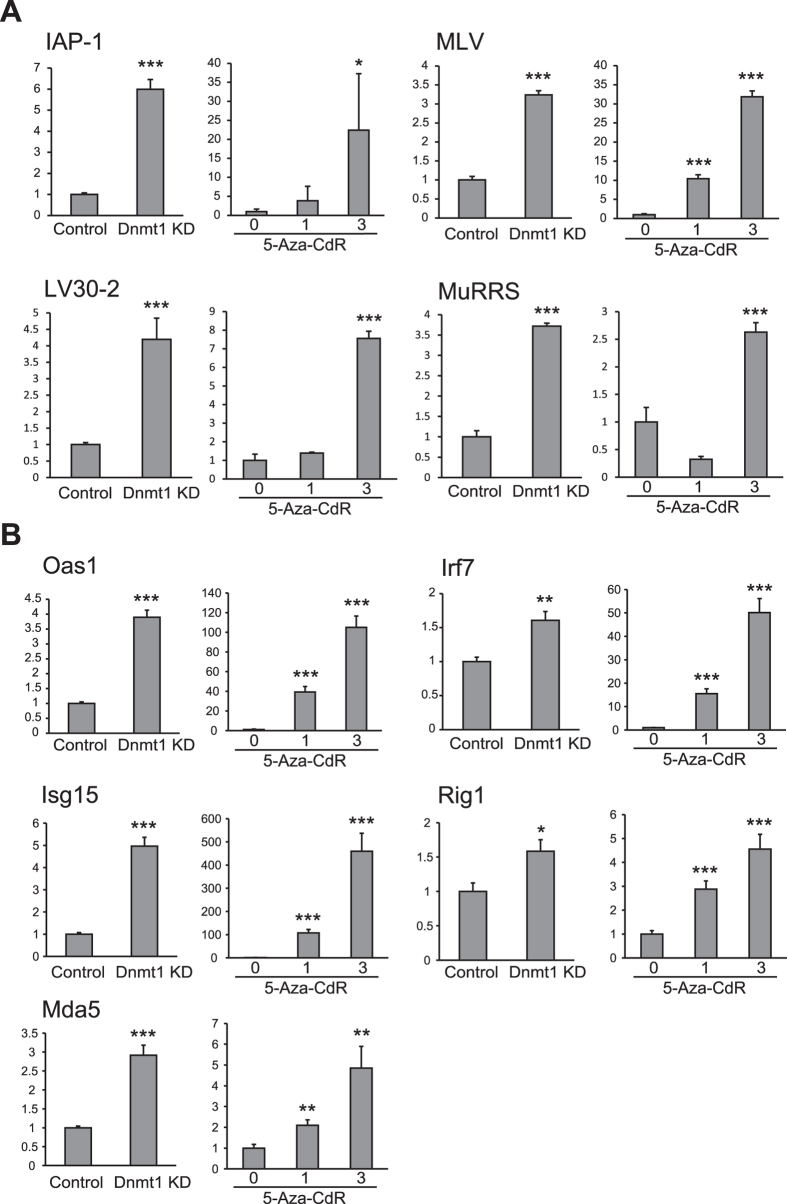
Expression levels of murine ERVs and interferon-responsive genes in intestinal tumor organoids after 5-Aza-CdR treatment and *Dnmt1* knockdown. (**A**) Relative expression levels of IAP-1, MLV, LV30-2 and MuRRS normalized to TBP in intestinal organoids after 5-Aza-CdR treatment (1 μM and 3 μM) and *Dnmt1* knockdown. *p < 0.05 and ***p < 0.0001 compared to control. (**B**) Relative expression levels of *Oas1, Irf7, Isg15, Rig1* and *Mda5* normalized to β*-actin* in intestinal organoids after 5-Aza-CdR treatment (1 μM and 3 μM) and *Dnmt1* knockdown. *p < 0.05, **p < 0.001 and ***p < 0.0001 compared to control.

To identify the genes that were differentially expressed upon inhibition of DNA methylation, we conducted microarray analyses of *Apc*-deficient intestinal tumor organoids after treatment with 1 μM 5-Aza-CdR and knockdown of *Dnmt1*. Total RNAs were extracted at 4 days after 1 μM 5-Aza-CdR treatment and at 9 days after the lentivirus infection, respectively. At these points, we confirmed that the growth of the organoids and *Dnmt1* expression were significantly decreased. A number of genes were significantly up-regulated in the organoids by pharmacologic and genetic inhibition of DNA methylation. Table 1 shows the top 25 genes that were commonly up-regulated by both 5-Aza-CdR treatment and *Dnmt1* knockdown in the tumor organoids. All data for the genes activated by DNA demethylation are available in Supplementary Table S2. As shown in Table 1, interferon-responsive genes including *2*′*-5*′ *oligoadenylate synthetase (Oas)* and *interferon regulatory factor 7 (Irf7)* were markedly activated in the tumor organoids after treatment with 5-Aza-CdR and knockdown of *Dnmt1*. Fifteen out of the top 25 up-regulated genes (60%) included the term “immune” or “interferon” in the gene name descriptor and GO annotation, indicating that these genes activated by DNA demethylation are involved in the pathway of the interferon and immune response. To determine whether the genes activated by DNA demethylation play a role in biological process, GO analysis was performed. The top 10 terms demonstrated by GO analysis of the genes commonly up-regulated by both 5-Aza-CdR treatment and *Dnmt1* knockdown in the tumor organoids are shown in Table 2. The GO analysis revealed that the genes that were activated by DNA demethylation in the tumor organoids are involved in the anti-viral response, immune response and cellular response to interferon and exogenous dsRNA. Moreover, pathway enrichment analysis also revealed that these genes up-regulated by DNA demethylation were associated with the KEGG pathways of the anti-viral responses to measles and hepatitis C (Table 2). Finally, to validate activation of the RIG-1/MDA5 RNA response pathway, we examined expression levels of interferon-responsive genes and murine ERVs in intestinal tumor organoids after 5-Aza-CdR treatment and *Dnmt1* knockdown. Expression levels of murine ERVs such as IAP-1, MLV, LV30-2 and MuRRS were significantly increased in the organoids after 5-Aza-CdR treatment and *Dnmt1* knockdown (Fig. 3A). Moreover, expression levels of interferon-responsive genes such as *Oas1, Irf7, Isg15, Rig1* and *Mda5* were also increased by 5-Aza-CdR treatment in a dose-dependent manner and *Dnmt1* knockdown (Fig. 3B).

## Discussion

Several studies have demonstrated that DNA methylation inhibitors such as 5-Aza-CdR and zebularine suppress intestinal tumors in *Min* mice^17^,^18^. However, the molecular mechanisms underlying this anti-tumor effect remain to be clarified. So far, the major mechanism responsible for the anti-tumor effect of DNA methylation inhibitors has been considered to be re-activation of silenced tumor suppressor genes by DNA demethylation of their CpG island promoters. Recently, however, two groups, Roulois *et al*. and Chiappinelli *et al.*, have demonstrated that DNA demethylating agents can induce a cell-autonomous immune activation response by stimulating the expression of dsRNA-containing ERVs^3^,^4^. This may represent a paradigm shift in our understanding of the anti-tumor mechanism of DNA demethylating agents.

In Rspo1-based 3D culture system, Lgr5-positive stem cells form ever-expanding organoids that retain their tissue identity. The tumor organoids derived from *Apc*-deficient tumors can be stably maintained in culture medium without Rspo1 and form cystic organoid structures, because Apc loss constitutively activates the Wnt signaling pathway in tumor cells. Our present results show that pharmacologic and genetic DNA demethylation by 5-Aza-CdR treatment and *Dnmt1* knockdown suppresses the proliferation of *Apc*-deficient intestinal tumor organoids. In addition, the data from microarray analyses clearly demonstrate that the majority of genes commonly up-regulated by 5-Aza-CdR treatment and *Dnmt1* knockdown are associated with the immune response to virus and dsRNAs and the interferon response. Indeed, expression levels of murine ERVs and interferon-responsive genes including the Rig1/Mda5 RNA response pathway were significantly increased after 5-Aza-CdR treatment and *Dnmt1* knockdown. We investigated the levels of DNA methylation around the promoter regions of these genes, including *Irf7*, by pyrosequencing in intestinal tumor organoids, but found no significant difference after 5-Aza-CdR treatment and *Dnmt1* knockdown (data not shown). Therefore, our present results strongly support the hypothesis that the main anti-tumor effect of DNA demethylating agents is induction of an anti-viral response via activation of dsRNA-containing ERVs^3^,^4^.

Taken together, these findings indicate that inhibition of DNA methylation suppresses intestinal tumor organoids by inducing a growth-inhibitory immune response, including activation of interferon-responsive genes, in response to ERVs. Treatment with DNA methylation inhibitors to activate a growth-inhibiting immune response could be an effective therapeutic approach for colon cancers. In the future, patient-derived tumor organoids may allow evaluation of sensitivity to DNA methylation inhibitors and personalized epigenetic therapy for cancer.

## Additional Information

**How to cite this article**: Saito, Y. *et al*. Inhibition of DNA Methylation Suppresses Intestinal Tumor Organoids by Inducing an Anti-Viral Response. *Sci. Rep*. **6**, 25311; doi: 10.1038/srep25311 (2016).

## Supplementary Material

Supplementary Information

## Figures and Tables

**Table 1:**
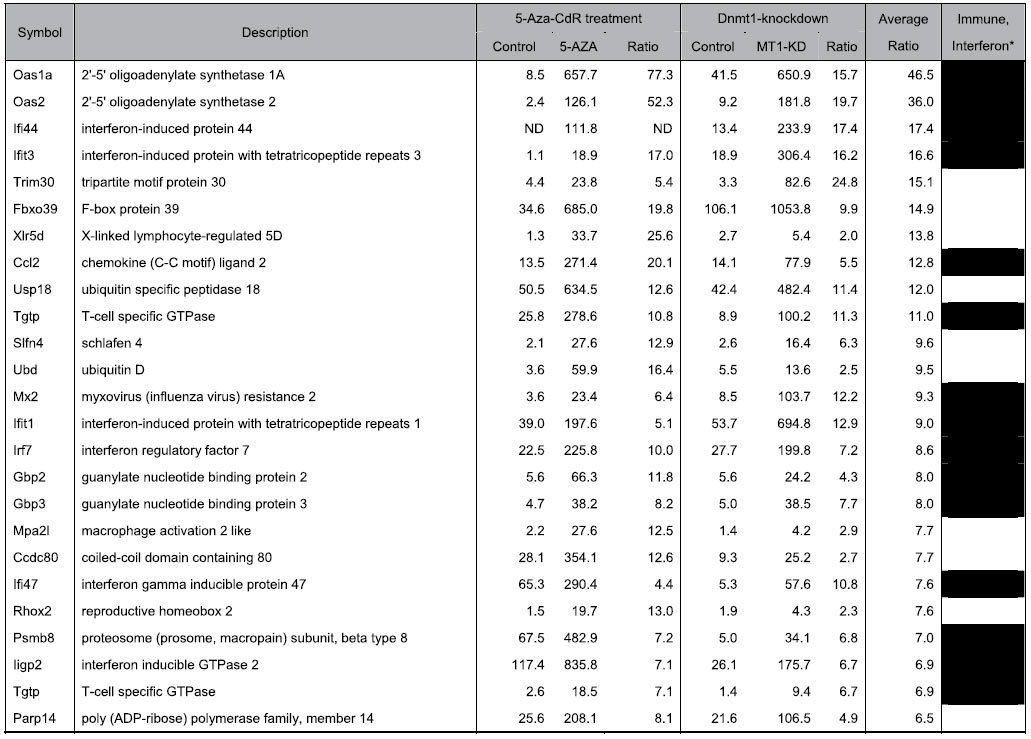
The top 25 genes that were commonly up-regulated by both 5-Aza-CdR treatment and Dnmt1-knockdown.

ND, not detected. *Genes that include the term “immune” or “interferon” in their description and gene ontology annotation are denoted by black.

**Table 2:** The GO and KEGG pathway analyses of the genes commonly up-regulated by both 5-Aza-CdR treatment and Dnmt1-knockdown.

Term	Description	Genes	NGR	NG	*p*-value
GO:0009615	Response to virus	Klra8,Zc3hav1,Ifih1,Irf7,Oas1b,Rsad2,Ifit1,Mx2	79 (36814)	8 (105)	2.36441E-08
GO:0035458	Cellular response to interferon-beta	Gbp3,Gbp2,Ifit3,Ifit1	17 (36814)	4 (105)	2.31915E-05
GO:0006955	Immune response	B2m,Oasl2,Oas1b,Ccl2,Oas1a,Ccl5	151 (36814)	6 (105)	0.00053118
GO:0071346	Cellular response to interferon-gamma	Gbp3,Gbp2,Ccl2	16 (36814)	3 (105)	0.000986171
GO:0019060	Intracellular transport of viral proteins in host cell	Tap1,Ifit1	4 (36814)	2 (105)	0.00309224
GO:0043123	Positive regulation of I-kappaB kinase/NF-kappaB cascade	Bst2,Rnf31,Zc3hav1,Ubd	98 (36814)	4 (105)	0.00535993
GO:0010759	Positive regulation of macrophage chemotaxis	Ccl2,Ccl5	7 (36814)	2 (105)	0.00538121
GO:0045071	Negative regulation of viral genome replication	Ifit1,Ccl5	7 (36814)	2 (105)	0.00538121
GO:0071360	Cellular response to exogenous dsRNA	Zc3hav1,Ifit1	7 (36814)	2 (105)	0.00538121
GO:0032020	ISG15-protein conjugation	Ube2l6,Usp18	6 (36814)	2 (105)	0.00550129
**Pathway**					
KEGG 05162	Measles	Mx2,Adar,Irf7,Stat2,Oas1a,Ifih1,Oas2,Oas1b	135 (36814)	8 (105)	2.85178E-07
KEGG 05160	Hepatitis C	Irf7,Stat2,Ifit1,Oas1a,Oas2,Oas1b	137 (36814)	6 (105)	7.35785E-05
KEGG 04623	Cytosolic DNA-sensing pathway	Ccl5,Adar,Irf7	62 (36814)	3 (105)	0.0130779
KEGG 04620	Toll-like receptor signaling pathway	Ccl5,Irf7,Cd80	100 (36814)	3 (105)	0.0387771

The top 10 terms of GO analysis of the genes commonly up-regulated by both 5-Aza-CdR treatment and Dnmt1-knockdown.

The KEGG Pathway analysis of the genes commonly up-regulated by both 5-Aza-CdR treatment and Dnmt1-knockdown.

GO, gene ontology; KEGG, Kyoto Encyclopedia of Genes and Genomes; NGR, Number of annotated genes in the reference list (Total number of genes in the reference list); NG, Number of annotated genes in the input list (Total number of genes in the input list).
