# Supplementary material for: Inhibition of DNA Methylation Suppresses Intestinal Tumor Organoids by Inducing an Anti-Viral Response
Source: Sci Rep. 2016 May 4;6:25311. doi: 10.1038/srep25311 (PMC4855203; doi:10.1038/srep25311)
Supplement: Supplementary Information [file srep25311-s1.pdf]

Supplementary Materials

Inhibition of DNA Methylation Suppresses Intestinal Tumor Organoids by Inducing an Anti-Viral Response

Yoshimasa Saito, Toshiaki Nakaoka, Kasumi Sakai, Toshihide Muramatsu, Kohta Toshimitsu, Masaki Kimura, Takanori Kanai, Toshiro Sato, Hidetsugu Saito

Supplementary Table S1
Primer sequences of murine ERVs and interferon-responsive genes

Primer	Sequence
IAP-1-forward	5'-GGAGAAGTCCTACCTCAGGGG-3'
IAP-1-reverse	5'-GGAGAACAAAATGTCATCCATATA-3'
MLV-forward	5'-CCAGACTTGATCCTGCTACA-3'
MLV-reverse	5'-CTCAGTCAGCCATCTCTGAC-3'
LV30-2-forward	5'-CTCGCACCTTTTCGCGCTCG-3'
LV30-2-reverse	5'-GCGAGGGGATCATCATAACA-3'
MuRRS-forward	5'-TTAGCCTCCTTCCGAGCTGA-3'
MuRRS-reverse	5'-GCCTCTGTCAGCCACCGTTT-3'
TBP-forward	5'-TTCTGAGAGCTCTGGAATTGTA-3'
TBP-reverse	5'-TGGATTGTTCTTCACTCTTGGC-3'
Oas1-forward	5'- ATTACCTCCTTCCCGACACC-3'
Oas1-reverse	5'-CAAACTCCACCTCCTGATGC-3'
Irf7-forward	5'-CTTCAGCACTTTCTTCCGAGA-3'
Irf7-reverse	5'-TGTAGTGTGGTGACCCTTGC-3'
Isg15-forward	5'-CCAGTCTCTGACTGTGAGAGC-3'
Isg15-reverse	5'-GCATCACTGTGCTGCTGGGAC-3'
Rig1-forward	5'-CCACCTACATCCTCAGCTACATGA-3'
Rig1-reverse	5'-TGGGCCCTTGTTGTTCTTCT-3'
Mda5-forward	5'-AGATCAACACCTGTGGTAACACC-3'
Mda5-reverse	5'-CTCTAGGGCCTCCACGAACA-3'
β-actin-forward	5'-GAAATAGTGCGTGACATCAAAG-3'
β-actin-reverse	5'-TGTAGTTTCATGGATGCCACAG-3'

Supplementary Table S2   The genes that were commonly up-regulated by both 5-Aza-CdR treatment and Dnmt1-knockdown

Symbol	Description	5-Aza-CdR treatment			Dnmt1-knockdown			Average Ratio
		Control	5-AZA	Ratio	Control	MT1-KD	Ratio	
2310016F22Rik	RIKEN cDNA 2310016F22 gene	2.1	262.9	122.9	8.1	152.6	18.9	70.9
Oas1a	2'-5' oligoadenylate synthetase 1A	8.5	657.7	77.3	41.5	650.9	15.7	46.5
Oas2	2'-5' oligoadenylate synthetase 2	2.4	126.1	52.3	9.2	181.8	19.7	36.0
Ifi44	interferon-induced protein 44	ND	111.8	ND	13.4	233.9	17.4	17.4
Ifit3	interferon-induced protein with tetratricopeptide repeats 3	1.1	18.9	17.0	18.9	306.4	16.2	16.6
Trim30	tripartite motif protein 30	4.4	23.8	5.4	3.3	82.6	24.8	15.1
Fbxo39	F-box protein 39	34.6	685.0	19.8	106.1	1053.8	9.9	14.9
Xlr5d	X-linked lymphocyte-regulated 5D	1.3	33.7	25.6	2.7	5.4	2.0	13.8
Oas1a	2'-5' oligoadenylate synthetase 1A	46.9	731.9	15.6	72.7	729.7	10.0	12.8
Ccl2	chemokine (C-C motif) ligand 2	13.5	271.4	20.1	14.1	77.9	5.5	12.8
Usp18	ubiquitin specific peptidase 18	50.5	634.5	12.6	42.4	482.4	11.4	12.0
Tgtp	T-cell specific GTPase	25.8	278.6	10.8	8.9	100.2	11.3	11.0
D12Ertd647e	ISG12a protein (DNA segment, Chr 12, ERATO Doi 647, expressed).	156.7	2664.2	17.0	187.1	568.8	3.0	10.0
Slfn4	schlafen 4	2.1	27.6	12.9	2.6	16.4	6.3	9.6
Ubd	ubiquitin D	3.6	59.9	16.4	5.5	13.6	2.5	9.5
Mx2	myxovirus (influenza virus) resistance 2	3.6	23.4	6.4	8.5	103.7	12.2	9.3
Ifit1	interferon-induced protein with tetratricopeptide repeats 1	39.0	197.6	5.1	53.7	694.8	12.9	9.0
Irf7	interferon regulatory factor 7	22.5	225.8	10.0	27.7	199.8	7.2	8.6
Gbp2	guanylate nucleotide binding protein 2	5.6	66.3	11.8	5.6	24.2	4.3	8.0
Gbp3	guanylate nucleotide binding protein 3	4.7	38.2	8.2	5.0	38.5	7.7	8.0
Mpa2l	macrophage activation 2 like	2.2	27.6	12.5	1.4	4.2	2.9	7.7
Ccdc80	coiled-coil domain containing 80	28.1	354.1	12.6	9.3	25.2	2.7	7.7
Ifi47	interferon gamma inducible protein 47	65.3	290.4	4.4	5.3	57.6	10.8	7.6
Rhox2	reproductive homeobox 2	1.5	19.7	13.0	1.9	4.3	2.3	7.6
BC013672	cDNA sequence BC013672	9.9	62.3	6.3	11.6	95.3	8.2	7.2
Psmb8	proteosome (prosome, macropain) subunit, beta type 8 (large multifunctional peptidase 7)	67.5	482.9	7.2	5.0	34.1	6.8	7.0
Iigp2	interferon inducible GTPase 2	117.4	835.8	7.1	26.1	175.7	6.7	6.9
Tgtp	T-cell specific GTPase	2.6	18.5	7.1	1.4	9.4	6.7	6.9
BC013672	cDNA sequence BC013672	6.4	23.6	3.7	16.2	159.9	9.9	6.8
Parp14	poly (ADP-ribose) polymerase family, member 14	25.6	208.1	8.1	21.6	106.5	4.9	6.5
Iigp2	interferon inducible GTPase 2	98.2	662.7	6.7	32.6	204.5	6.3	6.5
D14Ertd668e	Activated spleen cDNA, RIKEN full-length enriched library, clone:F830031K02 product:PHD finger protein 11, full insert sequence.	20.4	127.8	6.3		42.9		6.3
Cd80	CD80 antigen	0.8	7.9	9.4	5.5	16.0	2.9	6.1
1700007K13Rik	RIKEN cDNA 1700007K13 gene	34.9	167.7	4.8	2.8	20.6	7.4	6.1
Parp12	poly (ADP-ribose) polymerase family, member 12	26.8	207.4	7.7	23.6	93.4	4.0	5.8
Rsad2	radical S-adenosyl methionine domain containing 2	44.5	162.7	3.7	84.8	661.2	7.8	5.7
Irgm	immunity-related GTPase family, M	131.6	872.0	6.6	188.3	904.9	4.8	5.7
Hsh2d	hematopoietic SH2 domain containing	1.3	8.8	6.7	3.9	16.4	4.2	5.5
Mpa2l	macrophage activation 2 like	1.9	6.6	3.4	1.2	8.7	7.5	5.4
Ifih1	interferon induced with helicase C domain 1	18.1	110.3	6.1	31.6	147.6	4.7	5.4
Bst2	bone marrow stromal cell antigen 2	102.4	695.8	6.8	82.7	300.3	3.6	5.2
Oasl2	2'-5' oligoadenylate synthetase-like 2	24.9	102.2	4.1	28.9	179.4	6.2	5.2
Slfn2	schlafen 2	49.6	210.1	4.2	51.8	303.3	5.9	5.0
BC020489	cDNA sequence BC020489	433.6	2036.2	4.7	118.8	636.0	5.4	5.0
Trim34	tripartite motif protein 34	14.8	91.5	6.2	11.0	42.5	3.9	5.0
Rtp4	receptor transporter protein 4	137.6	667.2	4.8	208.8	1003.0	4.8	4.8
Oasl1	2'-5' oligoadenylate synthetase-like 1	49.2	271.7	5.5	166.9	680.4	4.1	4.8
Adar	adenosine deaminase, RNA-specific	12.1	77.8	6.5	16.4	51.5	3.1	4.8
Oas1b	2'-5' oligoadenylate synthetase 1B	3.8	17.6	4.7	2.9	14.3	4.9	4.8
Serpine2	serine (or cysteine) peptidase inhibitor, clade E, member 2	26.1	160.3	6.1	19.0	62.7	3.3	4.7
D11Ertd759e	13 days embryo male testis cDNA, RIKEN full-length enriched library, clone:6030403J01 product:similar to THYRO1000270 PROTEIN.	137.7	661.6	4.8	129.0	579.6	4.5	4.6
Cpeb1	cytoplasmic polyadenylation element binding protein 1	1.2	8.0	6.4	2.3	6.2	2.7	4.6
Parp9	poly (ADP-ribose) polymerase family, member 9	48.2	250.9	5.2	53.5	211.7	4.0	4.6
1810036H07Rik	RIKEN cDNA 1810036H07 gene	1.0	4.9	5.1	2.4	9.4	4.0	4.5
Vcam1	vascular cell adhesion molecule 1	4.7	27.0	5.8	16.5	54.4	3.3	4.5
Cd244	CD244 natural killer cell receptor 2B4	10.0	69.1	6.9	5.1	10.3	2.0	4.5
Tyki	thymidylate kinase family LPS-inducible member	13.9	64.1	4.6	11.7	49.8	4.3	4.4
1700003E16Rik	RIKEN cDNA 1700003E16 gene	1.9	11.7	6.3	1.1	2.8	2.5	4.4
Isgf3g	interferon dependent positive acting transcription factor 3 gamma	51.6	286.2	5.5	86.7	277.9	3.2	4.4
2200002D01Rik	RIKEN cDNA 2200002D01 gene	8.4	51.1	6.1	67.1	165.3	2.5	4.3
Iigp2	interferon inducible GTPase 2	206.7	674.7	3.3	10.7	55.7	5.2	4.2
2810022L02Rik	RIKEN cDNA 2810022L02 gene	3.4	14.6	4.3	9.1	37.9	4.2	4.2
Psmb9	proteosome (prosome, macropain) subunit, beta type 9 (large multifunctional peptidase 2)	77.8	234.5	3.0	11.7	62.2	5.3	4.2
Rasgrf2	RAS protein-specific guanine nucleotide-releasing factor 2	0.9	4.8	5.2	1.6	4.8	3.0	4.1
D11Lgp2e	Probable ATP-dependent helicase LGP2 (EC 3.6.1.-) (Protein D11Lgp2).	18.0	90.2	5.0	14.9	46.5	3.1	4.1
Oas1c	2'-5' oligoadenylate synthetase 1C	7.9	43.1	5.5	6.5	16.6	2.6	4.0
Ak1	adenylate kinase 1	14.1	77.3	5.5	18.7	47.1	2.5	4.0
B2m	beta-2 microglobulin	3312.5	14584.6	4.4	1191.3	4267.8	3.6	4.0
Dtx3l	deltex 3-like (Drosophila)	37.3	189.3	5.1	40.2	113.0	2.8	3.9
Ccl5	chemokine (C-C motif) ligand 5	10.1	38.2	3.8	8.4	32.7	3.9	3.8
Il18bp	interleukin 18 binding protein	2.3	12.6	5.4	3.2	7.1	2.2	3.8
Sncg	synuclein, gamma	19.9	88.2	4.4	8.7	27.6	3.2	3.8
Parp14	poly (ADP-ribose) polymerase family, member 14	1.3	5.0	3.8	4.0	14.1	3.6	3.7
Apof	apolipoprotein F	3.4	14.5	4.3	1.1	3.1	2.8	3.5
Tnfrsf18	tumor necrosis factor receptor superfamily, member 18	24.6	97.1	3.9	33.0	94.3	2.9	3.4
Zfp750	zinc finger protein 750	14.0	62.2	4.4	22.3	53.0	2.4	3.4
Trim12	tripartite motif protein 12	37.4	133.5	3.6	30.5	95.2	3.1	3.3
Npb	neuropeptide B	4.3	18.2	4.3	1.2	2.9	2.4	3.3
Tap1	transporter 1, ATP-binding cassette, sub-family B (MDR/TAP)	30.7	136.1	4.4	26.6	57.7	2.2	3.3
Timp1	tissue inhibitor of metalloproteinase 1	10.5	35.6	3.4	1.3	4.1	3.2	3.3
BC057170	cDNA sequence BC057170	7.5	21.9	2.9	4.3	15.8	3.7	3.3
E330016A19Rik	RIKEN cDNA E330016A19 gene	1.7	4.3	2.5	1.8	7.2	4.1	3.3
Ifi35	interferon-induced protein 35	107.0	315.5	2.9	92.7	333.2	3.6	3.3
Stat2	signal transducer and activator of transcription 2	24.1	75.3	3.1	28.3	94.7	3.3	3.2
Scn1b	sodium channel, voltage-gated, type I, beta	4.1	15.7	3.9	1.3	3.2	2.5	3.2
NP_001020554.1	similar to melanoma associated antigen (mutated) 1-like 1 (LOC674060), mRNA	1.2	4.7	3.8	4.2	10.4	2.5	3.1
Ube2l6	ubiquitin-conjugating enzyme E2L 6	71.2	168.2	2.4	34.6	129.3	3.7	3.0
Fcrl1	Fc receptor-like 1	2.5	6.3	2.5	1.1	3.8	3.4	3.0
Ogfr	opioid growth factor receptor	182.8	604.1	3.3	112.5	290.3	2.6	2.9
BC057170	cDNA sequence BC057170	30.7	97.5	3.2	29.0	78.1	2.7	2.9
Slc36a3	solute carrier family 36 (proton/amino acid symporter), member 3	1.4	3.1	2.1	1.2	4.3	3.6	2.8
Krt5	keratin 5	2.5	7.7	3.0	3.0	7.9	2.6	2.8
OTTMUSG00000000651	predicted gene, OTTMUSG00000000651	2.5	8.3	3.3	28.6	64.0	2.2	2.8
Npnt	nephronectin	1.0	3.5	3.4	1.7	3.6	2.1	2.8
Casp4	caspase 4, apoptosis-related cysteine peptidase	60.9	208.8	3.4	172.7	358.1	2.1	2.8
Olfr360	olfactory receptor 360	1.2	4.1	3.3	2.6	5.8	2.2	2.7
D330028D13Rik	RIKEN cDNA D330028D13 gene	6.0	14.8	2.5	2.1	6.0	2.9	2.7
Mkx	mohawk	5.6	11.3	2.0	36.5	120.1	3.3	2.6
Foxj1	forkhead box J1	4.7	15.2	3.2	10.0	20.9	2.1	2.6
Gap43	growth associated protein 43	1.2	2.6	2.1	1.3	4.1	3.2	2.6
Rnf31	ring finger protein 31	51.9	143.4	2.8	24.3	58.7	2.4	2.6
Rnf26	ring finger protein 26	3.4	8.8	2.6	1.8	4.7	2.6	2.6
Plxdc2	plexin domain containing 2	3.2	10.0	3.1	2.0	4.0	2.0	2.6
EG330552	predicted gene, EG330552	1.2	3.6	2.9	2.4	5.5	2.3	2.6
Ankrd29	ankyrin repeat domain 29	5.4	14.6	2.7	4.9	11.9	2.4	2.6
St3gal2	ST3 beta-galactoside alpha-2,3-sialyltransferase 2	14.1	42.3	3.0	11.7	24.5	2.1	2.5
Tnfrsf17	tumor necrosis factor receptor superfamily, member 17	1.4	3.0	2.1	1.8	5.3	3.0	2.5
Zc3hav1	zinc finger CCCH type, antiviral 1	50.0	121.2	2.4	32.7	86.0	2.6	2.5
Nmi	N-myc (and STAT) interactor	158.9	425.4	2.7	98.1	232.7	2.4	2.5
S100a9	S100 calcium binding protein A9 (calgranulin B)	0.8	1.9	2.5	35.7	92.5	2.6	2.5
Prss28	protease, serine, 28	1.1	3.0	2.7	2.6	6.0	2.3	2.5
9230105E10Rik	RIKEN cDNA 9230105E10 gene	7.1	17.3	2.4	11.3	29.2	2.6	2.5
Grb10	growth factor receptor bound protein 10	1.7	4.3	2.5	2.4	6.0	2.5	2.5
Hpcal4	hippocalcin-like 4	2.0	5.9	2.9	1.6	3.3	2.1	2.5
Plec1	plectin 1	93.7	209.9	2.2	79.2	215.5	2.7	2.5
Sec31b	Sec31 homolog B (S. cerevisiae)	1.0	2.5	2.4	1.5	3.8	2.6	2.5
Trim21	tripartite motif protein 21	21.3	46.7	2.2	22.3	60.6	2.7	2.5
Rnf182	ring finger protein 182	2.9	6.1	2.1	1.9	5.0	2.7	2.4
ENSMUSG00000060719	predicted gene, ENSMUSG00000060719	0.9	2.0	2.2	1.6	4.1	2.5	2.3
Ipo11	importin 11	0.8	2.2	2.6	3.0	6.2	2.1	2.3
Wdr85	WD40 repeat domain 85	1.3	3.3	2.5	10.2	21.5	2.1	2.3
Krt12	keratin 12	1.6	4.1	2.6	6.4	13.1	2.1	2.3
Tor3a	torsin family 3, member A	60.4	157.8	2.6	67.9	136.1	2.0	2.3
Sart2	squamous cell carcinoma antigen recognized by T cells 2	1.0	2.5	2.4	2.0	4.4	2.2	2.3
3100002H09Rik	RIKEN cDNA 3100002H09 gene	1.6	3.4	2.2	4.8	11.6	2.4	2.3
A330084C13Rik	RIKEN cDNA A330084C13 gene	1.1	2.7	2.4	4.4	9.4	2.1	2.3
2310002J15Rik	RIKEN cDNA 2310002J15 gene	15.9	37.3	2.3	65.3	142.9	2.2	2.3
Klra8	killer cell lectin-like receptor, subfamily A, member 8	1.2	2.8	2.4	1.8	3.8	2.2	2.3
Wfikkn1	WAP, FS, Ig, KU, and NTR-containing protein 1	6.4	15.4	2.4	2.7	5.6	2.1	2.2
Rassf1	Ras association (RalGDS/AF-6) domain family 1	85.9	176.8	2.1	117.9	283.3	2.4	2.2
NP_001033765.1	similar to oocyte specific homeobox 3 (MGC117731), mRNA	2.5	5.2	2.1	2.7	6.5	2.4	2.2
N28178	expressed sequence N28178	2.3	5.2	2.3	1.5	3.3	2.2	2.2
Kcnh6	potassium voltage-gated channel, subfamily H (eag-related), member 6	14.1	33.5	2.4	123.1	252.1	2.0	2.2
Hsf2	heat shock factor 2	3.0	6.2	2.0	1.2	2.9	2.4	2.2
EG384814	predicted gene, EG384814	1.4	3.2	2.3	1.8	3.6	2.0	2.2
Twf2	twinfilin, actin-binding protein, homolog 2 (Drosophila)	159.2	350.1	2.2	88.2	185.4	2.1	2.2
Gadd45b	growth arrest and DNA-damage-inducible 45 beta	278.4	575.0	2.1	223.5	499.7	2.2	2.2
4933407L21Rik	RIKEN cDNA 4933407L21 gene	1.9	4.1	2.1	1.8	4.0	2.2	2.2
Speer8-ps1	spermatogenesis associated glutamate (E)-rich protein 8, pseudogene 1	1.4	2.9	2.1	7.1	15.7	2.2	2.1
Parp11	poly (ADP-ribose) polymerase family, member 11	15.6	33.8	2.2	12.8	26.2	2.1	2.1
Frmd5	FERM domain containing 5	1.2	2.5	2.1	1.4	3.0	2.1	2.1
Slc1a3	solute carrier family 1 (glial high affinity glutamate transporter), member 3	2.4	5.2	2.2	4.5	9.1	2.0	2.1
Ntng1	netrin G1	7.1	15.0	2.1	1.9	3.7	2.0	2.1
Tsen54	tRNA splicing endonuclease 54 homolog (SEN54, S. cerevisiae)	139.7	279.7	2.0	184.7	380.5	2.1	2.0
